# Coffee Intake, Plasma Caffeine Levels, and Kidney Function: Two-Sample Mendelian Randomization Among East Asian and European Ancestries

**DOI:** 10.1016/j.ekir.2024.01.024

**Published:** 2024-01-19

**Authors:** Ryosuke Fujii, Masahiro Nakatochi, Fabiola Del Greco M.

**Affiliations:** 1 Institute for Biomedicine (affiliated to the University of Lübeck), Eurac Research, Bolzano/Bozen, Italy; 2 Department of Preventive Medical Science, Fujita Health University School of Medical Sciences, Toyoake, Japan; 3 Department of Preventive Medicine, Nagoya University Graduate School of Medicine, Nagoya, Japan; 4 Public Health Informatics Unit, Department of Integrated Health Sciences, Nagoya University Graduate School of Medicine, Nagoya, Japan

**Keywords:** caffeine, CKD, coffee, eGFR, kidney function, mendelian randomization

## Abstract

**Introduction:**

Previous Mendelian randomization (MR) studies for the coffee-kidney association have reported inconsistent relationships in European populations and never examined mediators of this association. We aimed to evaluate this causal relationship using two-sample MR among both East Asian and European ancestries and to explore underlying mechanisms using plasma caffeine levels.

**Methods:**

Among East Asians, the largest genome-wide association study (GWAS) results for coffee intake, plasma caffeine levels, and kidney outcomes were obtained from 152,634; 8940; and 47,070 Japanese adults. Among Europeans, summary statistics were acquired from European GWAS with 428,860; 7719; and 564,470 adults for each trait. We applied different MR methods (inverse-variance weighted [IVW] with random effects, weighted median, weighted mode, and MR-Egger).

**Results:**

After excluding possible pleiotropic variants, among East Asian ancestry, drinking an extra coffee intake per week showed a protective association on serum creatinine-based estimated glomerular filtration rate (eGFRcre) (*β* = 0.077; 95% confidence interval [CI] = 0.003 to 0.150). Analysis in European ancestry also showed a causal relationship between drinking an extra coffee intake per day and eGFRcre (*β* = 0.052; 95% CI = 0.027 to 0.078). These results were consistent across different MR methods accounting for invalid instruments. Higher plasma caffeine levels were associated with lower eGFRcre among both East Asian (*β* = −0.071; 95% CI = −0.137 to −0.006) and European ancestries (*β* = −0.048; 95% CI = –0.057 to −0.040).

**Conclusions:**

Our cross-ancestry MR study found beneficial effects of coffee intake on eGFRcre. However, given the possible adverse effects of plasma caffeine levels on eGFRcre, interpretation of the results should be carefully considered and further investigations on noncaffeine and biological pathways are needed.

## Graphical abstract

**Figure:**
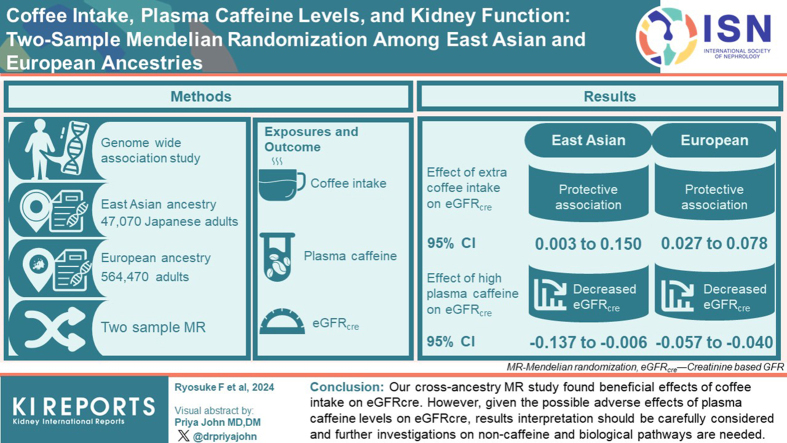

Chronic kidney disease (CKD) is one of the major health concerns with a prevalence of >10% worldwide.^1^ CKD is estimated to be the fifth cause of death by 2040.^2^ Nevertheless, molecular mechanisms underlying progressive renal dysfunction are largely unknown, resulting in a lack of ideal therapeutic medication. Thus, at this time, establishing a public health approach will play a key role in reducing CKD cases.

Coffee is a commonly consumed beverage worldwide and an important modifiable factor in public health intervention.^3^ Some previous epidemiological studies also supported that habitual coffee intake may have a protective role on mortality and other diseases across different regions and clinical conditions.^4^, ^5^, ^6^, ^7^ In nephrology, evidence for drinking coffee on kidney function are mostly beneficial^8^, ^9^, ^10^, ^11^, ^12^; however, there are still inconclusive discussions with null^13^ or negative directions.^14^ Another concern in these observational studies could introduce a bias in the estimates by potential confounding factors (e.g., hypertension and diabetes) and reverse causality (e.g., declined kidney function discourages coffee consumption).

MR is an emerging statistical methodology to infer causal relationships between modifiable exposure and outcome even in observational studies.^15^ This method is an analogous approach to randomized clinical trials by using genetic variants as instrumental variables (IVs). Regarding coffee-kidney causal associations, MR could be the best way to address this question because this method could address limitations in observational studies mentioned above. Two previous MR studies examined whether genetically-determined coffee intake could be causally associated with different kidney traits using genetic studies among trans-ethnic (mostly Europeans) and European ancestries.^16^^,^^17^ However, 3 limitations are still left to be addressed. To begin with, the results were inconsistent between these 2 previous studies. In addition, these studies did not address the underlying mechanism of the coffee-kidney relationship (i.e., caffeine and other risk factors). Moreover, both studies were conducted using summary statistics of GWAS from mainly European ancestries. Given different lifestyles and genetic backgrounds in every ancestry, it should be valuable to investigate this association among non-European ancestries.

Here, our primary purpose is to evaluate whether coffee consumption has a potential association with kidney function in both East Asian and European ancestries. The secondary purpose is to explore the mechanisms underlying this relationship using plasma caffeine levels.

## Methods

**Figure 1:**
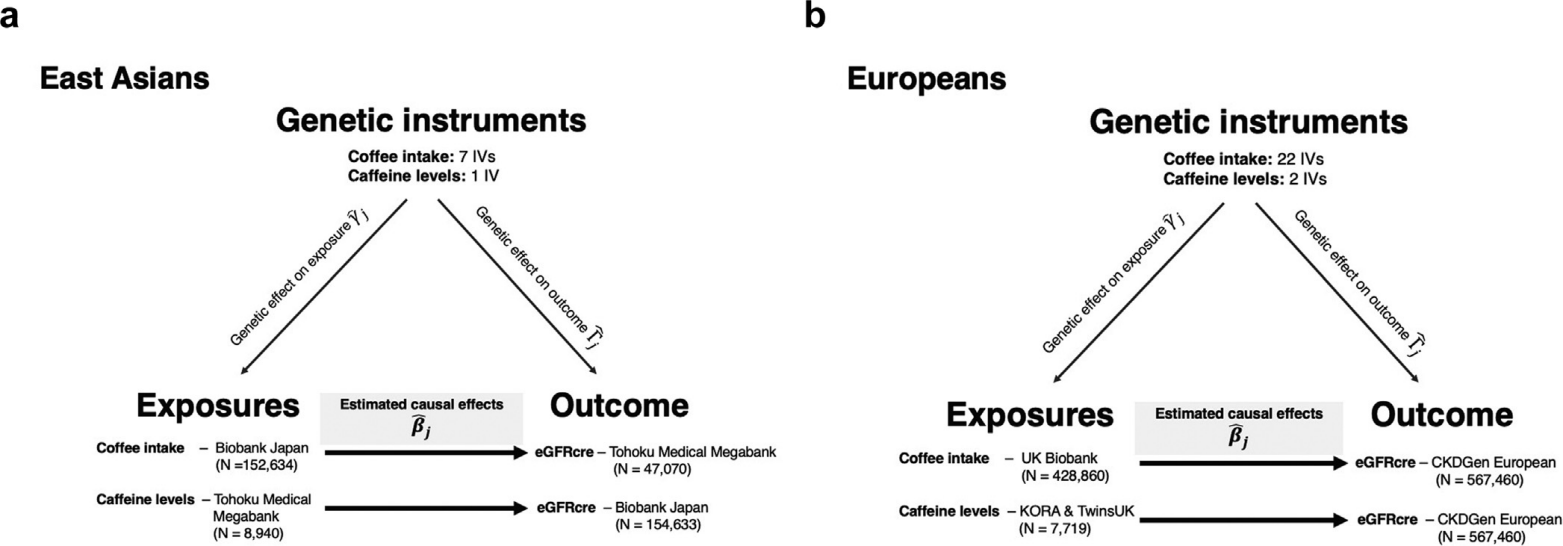
Outline and information of our Mendelian randomization analysis. eGFR, serum creatinine-based estimated glomerular filtration rate.

An Overview of our MR analysis is shown in Figure 1. More detailed information on GWAS summary statistics (coffee intake, plasma caffeine levels, and kidney function) from East Asian, only including Japanese GWAS because of nature of sample size, and European ancestries is described below and tabulated in Supplementary Table S1.

### Summary Data for Coffee Intake, Caffeine Levels, and Kidney Function From East Asian Ancestry

Summary data for coffee consumption in East Asian populations were obtained from a study on food groups and beverage types conducted by Biobank Japan.^18^ This GWAS was performed among 152,634 Japanese adults (https://pheweb.jp/pheno/Coffee). Regarding the outcome, it is defined as follows: 0 = rarely; 1.5 = 1 to 2 times per week; 3.5 = 3 to 4 times per week; 7 = almost every day. In other words, a 1-unit change in the genetic variant-exposure effect size indicates “drink an extra coffee intake per week.”

Summary data for kidney function were obtained from the Tohoku Medical Megabank Organization, which is organized by Tohoku University.^19^ Data and biological samples were collected from residents in 2 prefectures (Miyagi and Iwate) soon after the 2011 Great East Japan Earthquake. The GWAS results are also publicly available (with pre-authentication by ORCID) on a portal site called jMorp (https://jmorp.megabank.tohoku.ac.jp/gwas-studies).^20^ In this study, we used GWAS results from 47,000 Japanese participants in the Tohoku Medical Megabank Project Community-based Cohort (TGA000007; https://jmorp.megabank.tohoku.ac.jp/gwas-studies/TGA000007). eGFRcre was estimated by the Chronic Kidney Disease Epidemiology Collaboration formulas modified for Japanese people.^21^ According to their description, a 1-unit change in the genetic variant-outcome effect size indicates “1 SD change in eGFR per allele”.

Summary data for caffeine levels were again obtained from Tohoku Medical Megabank Organization. They also performed plasma metabolome GWAS among 8940 Japanese participants. The results and details for metabolome GWAS are available and described in the jMorp (TGA000005; https://jmorp.megabank.tohoku.ac.jp/gwas-studies/TGA000005). A 1-unit change in the genetic variant-outcome effect size indicates “1 SD change in caffeine levels.”

Given that we acquired the summary data for caffeine levels from Tohoku Medical Megabank Organization, it is required to find a GWAS result from an independent population for a two-sample MR analysis for caffeine-kidney function. In our MR analysis for the caffeine-kidney association, summary data for kidney function was obtained from the Biobank Japan website (https://pheweb.jp/pheno/eGFR) with 154,633 Japanese populations.^22^ One-unit change in the genetic variant-outcome effect size indicates “1 SD change in eGFR per allele.”

### Summary Data for Coffee Intake, Caffeine Levels, and Kidney Function From European Ancestry

For summary data on coffee intake, we acquired the dataset from a website of the IEU Open GWAS project (https://gwas.mrcieu.ac.uk/datasets/).^23^ Summary data were derived from the UK Biobank with 428,860 females and males. In the UK Biobank, from many questions about coffee beverage intake (decaf coffee, instant coffee, etc.), the general coffee intake (field ID: 1498) was selected as a proxy of the exposure in this study. We obtained summary statistics of coffee intake (ID: ukb-b-5237) from the GWAS Catalog database (https://www.ebi.ac.uk/gwas/).^24^ A 1-unit change in the genetic variant-exposure association indicates “drink an extra cup of coffee per day.”

For summary statistics on caffeine levels, we accessed the GWAS Catalog too with the study accession ID: GCST90243411 and downloaded a GWAS results for plasma metabolites in 7719 European individuals from 2 different studies (KORA F4, Germany and TwinsUK, United Kingdom).^25^ The details of this GWAS were described in Supplementary materials in their paper.

Summary statistics on kidney function (eGFRcre) were obtained from the CKDGen consortium website (https://ckdgen.imbi.uni-freiburg.de/). For eGFRcre, a meta-analysis of CKDGen Round 4 was performed among 567,460 in only European populations.^26^ eGFRcre values were estimated with the Chronic Kidney Disease Epidemiology Collaboration formula.^27^^,^^28^ Log-transformed eGFR values were regressed on sex and age, and its regression residuals were regressed on genetic variant dosage. Therefore, 1-unit change in the genetic variant-outcome association indicates “1 SD change in log(eGFR).”

### Selection of Genetic Instruments for East Asians

For coffee intake, 11 genetic variants were identified in the previous GWAS and selected as IVs (Table 1). All IVs were associated with exposure (*P* < 5.0 × 10^−8^ and *F*-statistic > 10) and uncorrelated, that is, not in linkage disequilibrium with a correlation coefficient *r*^2^ < 0.01. Linkage disequilibrium between genetic variants within the same chromosome was checked using the SNPclip Tool (https://ldlink.nci.nih.gov/?tab=snpclip). However, 5 IVs might introduce a bias in MR estimates due to heterogeneity, but 1 IV (rs4410790) has no pleiotropic effect with other traits. After excluding 4 IVs responsible for significant heterogeneity, 7 IVs were finally included as robust IVs of coffee intake in our two-sample MR analyses (bold texts in Table 1).

**Table 1:** Eleven genetic variants associated with coffee intake in a GWAS among East Asian ancestry^a^

Variant ID	Chr:Position^b^	Nearest gene	A1/A2^c^	AF(A1)	Beta	SE	*P*
**rs6681426**	1:150586971	*MCL1*	A/G	0.656	−0.078	0.012	1.06 × 10^−10^
rs1260326	2:27730940	*GCKR*	T/C	0.56	−0.096	0.011	9.86 × 10^−17^
**rs75544042**	4:89045331	*ABCG2*	G/A	0.703	0.069	0.012	4.89 × 10^−8^
rs12189679	6:98333409	*MIR2113*	G/A	0.637	−0.067	0.012	2.53 × 10^−8^
**rs13234378**	7:73026151	*MLXIPL*	A/T	0.896	−0.108	0.019	1.58 × 10^−8^
**rs3815455**	7:75611756	*POR*	C/T	0.583	−0.067	0.011	1.12 × 10^−8^
**rs4410790**	7:17284577	*AHR*	T/C	0.63	−0.213	0.012	8.08 × 10^−68^
rs662799	11:116663707	*APOE5*	A/G	0.646	0.066	0.012	4.25 × 10^−8^
rs671	12:112241766	*ALDH2*	G/A	0.75	−0.354	0.101	3.08 × 10^−153^
**rs58806801**	15:75059546	*CYP1A2,CSK*	G/A	0.777	0.084	0.014	2.43 × 10^−9^
**rs5760444**	22:24878218	*ADORA2A-AS1*	C/T	0.589	−0.073	0.011	3.70 × 10^−10^

AF, allele frequency; Chr, chromosome; SE, standard error.

a Seven genetic variants with bold fonts were used in final analysis after excluding variants responsible for heterogeneity.

b Position is based on CRGh37.

c A1 indicates effect allele, while A2 for the other allele.

For caffeine levels, there was no genome-wide significant genetic variant in a Japanese population. Based on European GWAS, we picked up 2 genetic variants (rs4410790 and rs2472297) as candidates for IV; however, rs2472297 is a rare variant in East Asian ancestry (minor allele frequency = 0.0008). rs4410790 was not genome-wide significant (*P* = 2.42 × 10^−4^), but associated with plasma caffeine levels (*F*-statistics > 10) and showed a consistent effect direction with European GWAS.

### Selection of Genetic Instruments for Europeans

For coffee intake, 40 genetic variants were selected as IVs. All IVs were associated with exposure (*P* < 5.0 × 10^−8^ and *F*-statistic > 10), but 10 were correlated with others in the same chromosome (linkage disequilibrium: *r*^2^ > 0.01) and excluded. In addition, 2 palindromic variants were excluded. Of the remaining 28 IVs, although 9 were detected as a potential bias in MR estimates only for analyses in eGFRcre, 3 genetic variants (rs4410790, rs2472297, and rs6062682) reported no pleiotropic effect in previous GWASs and directly related to coffee and caffeine metabolism. After excluding 6 IVs responsible for significant heterogeneity, 22 variants were finally included as robust IVs of coffee intake in European ancestry (Table 2).

**Table 2:** Twenty-eight genetic variants associated with coffee intake in a GWAS among European ancestry^a^

Variant ID	Chr:Position^b^	Nearest gene	A1/A2^c^	AF(A1)	Beta	SE	*P* value
**rs4615895**	1:96274668	*RP11-286B14.1*	A/G	0.741	0.012	0.002	4.20 × 10^−11^
**rs12989746**	2:49368391	*FSHR*	T/G	0.250	0.010	0.002	2.80 × 10^−8^
rs13387939	2:637498	*TMEM18*	A/C	0.828	0.017	0.002	9.80 × 10^−15^
**rs1527961**	2:62780440	*PSAT1P2*	C/T	0.135	−0.013	0.002	1.70 × 10^−8^
rs780093	2:27742603	*GCKR*	C/T	0.616	0.013	0.002	1.00 × 10^−15^
**rs2189234**	4:106075498	*TET2*	G/T	0.618	0.010	0.002	1.80 × 10^−9^
**rs13163336**	5:87943710	*LINC00461*	A/C	0.158	0.015	0.002	1.30 × 10^−11^
**rs2465037**	6:51179260	*RP11-228O6.2*	A/C	0.343	−0.011	0.002	4.80 × 10^−10^
rs9398171	6:108983527	*FOXO3*	T/C	0.711	0.011	0.002	1.10 × 10^−9^
rs34060476	7:73037956	*MLXIPL*	G/A	0.134	0.018	0.002	7.50 × 10^−15^
**rs4410790**	7:17284577	*AHR*	C/T	0.632	0.039	0.002	1.20 × 10^−120^
**rs7811609**	7:32930597	*KBTBD2*	T/C	0.375	0.009	0.002	4.00 × 10^−8^
**rs442355**	8:109128653	*AP001331.1*	C/G	0.254	−0.011	0.002	1.90 × 10^−9^
**rs6469262**	8:110443480	*PKHD1L1*	C/T	0.565	−0.009	0.002	1.90 × 10^−8^
**rs78267637**	8:33790200	*LOC105379364*	G/C	0.038	−0.025	0.004	3.90 × 10^−9^
**rs61928609**	12:11316437	*PRH1*	C/A	0.835	−0.015	0.002	1.30 × 10^−11^
**rs117968677**	15:75174251	*MPI*	A/G	0.024	−0.031	0.006	1.90 × 10^−8^
**rs2472297**	15:75027880	*CYP1A1-CYP1A2*	T/C	0.263	0.046	0.002	1.10 × 10^−142^
rs1421085	16:53800954	*FTO*	C/T	0.404	0.019	0.002	1.70 × 10^−29^
**rs57918684**	17:60150383	*MED13*	A/G	0.155	0.013	0.002	8.69 × 10^−9^
**rs7224815**	17:17845800	*TOM1L2*	T/A	0.408	−0.011	0.002	3.70 × 10^−11^
**rs1942965**	18:55032486	*ST8SIA3*	C/T	0.505	−0.009	0.002	3.80 × 10^−8^
rs476828	18:57852587	*MC4R*	C/T	0.237	0.017	0.002	5.60 × 10^−20^
**rs630194**	18:40950954	*SYT4*	C/T	0.343	−0.011	0.002	2.30 × 10^−11^
**rs75347775**	19:18495908	*GDF15,MIR3189*	A/G	0.245	0.010	0.002	2.70 × 10^−8^
**rs6062682**	20:62891820	*PCMTD2*	T/C	0.465	0.010	0.002	2.50 × 10^−10^
**rs6063085**	20:45840459	*ZMYND8*	C/A	0.373	0.010	0.002	4.50 × 10^−10^
**rs13054099**	22:41215672	*SLC25A17*	C/T	0.261	−0.011	0.002	4.30 × 10^−9^

AF, allele frequency; Chr, chromosome; SE, standard error.

a Twenty-two genetic variants with bold fonts were used in final analysis after excluding variants responsible for heterogeneity.

b Position is based on CRGh37.

c A1 indicates effect allele, while A2 for the other allele.

For caffeine levels, a meta-analysis of 7719 participants identified 2 variants near *AHR* (rs4410790; *P* = 4.03 × 10^−13^) and *CYP1A2* (rs2472297; *P* = 1.50 × 10^−10^). These variants were located in different chromosomes and associated with exposure (*F*-statistic > 10). Therefore, we selected these 2 variants as IVs for plasma caffeine intake. A recent MR regarding plasma caffeine levels also used the same 2 variants as IVs.^29^

### Two-sample MR analyses

We performed MR analyses using 3 different methods. The IVW method based on random effects is a conventional method to estimate causal relationships using summarized data in two-sample MR analysis, assuming a possible presence of balance pleiotropy.^30^ This method can meta-analyze ratio estimates ($\hat{\beta }$) between genetic variant-exposure (G-X; $\hat{\gamma }$) and genetic variant-outcome (G-Y; $\hat{\Gamma }$) associations with inverse-variance weights, accounting for possible heterogeneity for each IVW. An analysis for a coffee-eGFRcre relationship was performed with a multiplicative random effect model, whereas a fixed effect model was used for a caffeine-eGFRcre relationship.

Three alternative MR methods (weighted median,^31^ weighted mode,^32^ and MR-Egger^33^) were also performed for sensitivity analysis for directional pleiotropy. Violation of exclusion restriction assumption, also known as horizontal pleiotropy, can introduce bias into IVW estimates. Weighted median, weighted mode, and MR-Egger methods were developed to account for the effect of horizontal pleiotropy. MR-Egger provides a causal estimate using weak instruments (some variants have pleiotropic effects) and performs a formal test for directional pleiotropy. Weighted median method can estimate consistent effect sizes when 50% of the IVs are invalid. In addition, to detect which variants can be a cause for horizontal pleiotropy, we used the MR Pleiotropy RESidual Sum and Outlier (MR-PRESSO) global test.^34^ For outlier variants identified in the MR-PRESSO global test, we manually checked whether these variants were associated with any other traits or diseases by using Phenoscanner v2 (http://www.phenoscanner.medschl.cam.ac.uk/).^35^^,^^36^

Lastly, a leave-1-out analyses was also run to explore a possible presence of pleiotropic IV.^37^ Five MR methods (IVW, weighted median, weighted mode, MR-Egger and the leave-1-out) were performed using the R package “TwoSampleMR” (https://mrcieu.github.io/TwoSampleMR/articles/introduction.html). The MR-PRESSO was performed using the R package “MRPRESSO” (https://github.com/rondolab/MR-PRESSO).

## Results

### Coffee-Kidney Association in East Asian Ancestry

**Figure 2:**
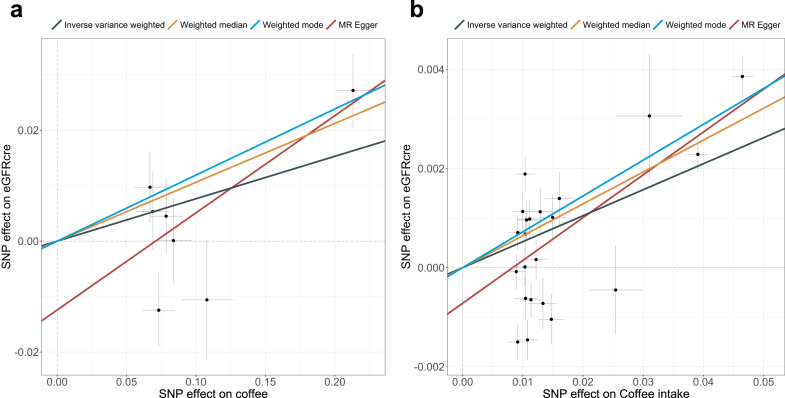
Scatter plot for variants associated with coffee intake against variants associated with eGFRcre among (a) East Asian and (b) European ancestry. Vertical and horizontal grey-colored solid lines around each point show 95% confidence intervals of estimates of variant-exposure and variant-outcomes associations. SNP, single nucleotide polymorphism.

The MR results using the original IV list (11 variants) are summarized in Supplementary Figures S1 to S3 and Supplementary Table S2. Although MR estimates with original IVs suggested coffee intake may increase the risk of kidney dysfunction, it is clearly biased by pleiotropic variants of rs1260326 (*GCKR*), rs671 (*ALDH2*), rs12189679 (*MIR2113*), and rs662799 (*APOE5*). With 7 rigorous variants, estimates from MR analyses are summarized in Table 3. In Figure 2a, we show a scatter plot for the variant-outcome associations (y-axis) against the variant-exposure associations (x-axis) based on 7 variants. Estimates from 4 MR methods are shown as solid straight lines with different slopes. An estimate from IVW shows strong evidence of an association between coffee intake and eGFRcre (*β* = 0.077; 95% CI = 0.003 to 0.150 SD in eGFRcre per coffee intake per week). We observed consistent estimates from weighted median (*β* = 0.107; 95% CI = 0.048 to 0.165), weighted mode (*β* = 0.119; 95% CI = 0.052 to 0.187), and MR-Egger (*β* = 0.175; 95% CI = 0.030 to 0.320). There is no evidence of directional pleiotropy based on MR-Egger intercepts (*P* = 0.19), but there is heterogeneity based on Cochran’s Q (Q = 14.6, *P* = 0.02). Forest plots for effect sizes of each variant on exposure and leave-out-one analysis are provided in Supplementary Figures S4 and S5.

**Table 3:** MR-estimated effect sizes of coffee-kidney function associations in East Asian and European ancestries

MR methods	East Asian		European	
	Beta (95% CI)^a^	*P* value	Beta (95% CI)^b^	*P* value
Inverse-variance weighted	0.077 (0.003, 0.150)	0.04	0.052 (0.027, 0.078)	5.70 × 10^−5^
Heterogeneity	Q = 14.6	0.02	Q = 140.4	1.14 × 10^−19^
Weighted median	0.107 (0.048, 0.165)	4.09 × 10^−4^	0.064 (0.048, 0.081)	1.90 × 10^−14^
Weighted mode	0.119 (0.052, 0.187)	0.01	0.072 (0.060, 0.085)	2.55 × 10^−10^
MR-Egger	0.175 (0.030, 0.320)	0.02	0.086 (0.043, 0.129)	8.04 × 10^−4^

CI, confidence interval; MR, Mendelian randomization.

a One-standard deviation change in eGFR per an extra cup of coffee intake per week

b One-standard deviation change in log(eGFR) per an extra cup of coffee per day

### Coffee-Kidney Association in European Ancestry

The MR results using the original IV list (28 genetic variants) are summarized in Supplementary Figure S6 to S8 and Supplementary Table S2. By using 22 robust genetic variants for eGFRcre, estimates from MR analyses are summarized in Table 3. In Figure 2b, we show a causal relationship of coffee intake with eGFRcre using European population GWAS summary statistics. An estimate from IVW shows strong evidence of an association between coffee and eGFRcre (*β* = 0.052; 95% CI = 0.027 to 0.078 log ml/min per 1.73 m^2^ per cup of coffee per day). Other MR methods also suggest a causal relationship between coffee consumption and eGFRcre in weighted median (*β* = 0.064; 95% CI = 0.048 to 0.081), weighted mode (*β* = 0.072; 95% CI = 0.060 to 0.085), and MR-Egger (*β* = 0.086; 95% CI = 0.043 to 0.129). Although there is no evidence of directional pleiotropy based on MR-Egger intercepts (*P* = 0.08), heterogeneity has still been left (Q = 140.4; *P* = 1.14×10^−19^) (Supplementary Figures S9 and S10).

### Plasma Caffeine-Kidney Association

In East Asian populations, GWAS results of rs4410790 across coffee intake, caffeine levels, and kidney function are summarized in Table 4. At rs4410790 (*AHR*), there are significant associations with plasma caffeine levels (*β* = −0.069; 95% CI = −0.105 to −0.032) and with eGFRcre (*β* = −0.0049; 95% CI = −0.0094 to −0.0003). Based on these summary statistics, IVW shows a causal association with genetically-determined plasma caffeine levels (*β* = −0.071; 95% CI = −0.137 to −0.006).

**Table 4:** Effect sizes of coffee intake, plasma caffeine levels, and eGFRcre for rs4410790 at *AHR* among East Asian ancestry

Phenotype	Variant ID	Chr:Position^a^	Nearest gene	A1/A2^b^	AF(A1)	Beta	SE	*P* value
Coffee intake	rs4410790	7:17284577	*AHR*	T/C	0.630	−0.2130	0.0119	8.08 × 10^−68^
Plasma caffeine	rs4410790	7:17284577	*AHR*	T/C	0.637	0.0686	0.0187	2.42 × 10^−4^
eGFRcre	rs4410790	7:17284577	*AHR*	T/C	0.622	−0.0049	0.0023	0.03

AF: allele frequency; Chr: chromosome; eGFRcre, serum creatinine-based estimated glomerular filtration rate; SE: standard error.

a Position is based on CRGh37.

b A1 indicates effect allele, while A2 for another allele.

By using summary statistics from European ancestries, the variant-exposure and the variant-outcome associations are summarized in Table 5. Significant relationships were observed in plasma caffeine levels at both rs4410790 in *AHR* (*β* = −0.063; 95% CI = −0.234 to −0.046) and rs2472297 in *CYP1A2* (*β* = −0.067; 95% CI = −0.087 to −0.046). The variant-outcome are also significant at both rs4410790 in *AHR* (*β* = 0.002; 95% CI = 0.002 to 0.003) and rs2472297 in *CYP1A2* (*β* = 0.004; 95% CI = 0.003 to 0.005). Based on these genetic variants, IVW shows a causal association with genetically-determined plasma caffeine levels (*β* = −0.048; 95% CI = −0.057 to −0.040).

**Table 5:** Effect sizes of coffee intake, plasma caffeine levels, and eGFRcre for two genetic variants (rs4410790 at *AHR* and rs2472297 at *CYP1A1*) among European ancestry

Phenotype	Variant ID	Chr:Position^a^	Nearest gene	A1/A2^b^	AF(A1)	Beta	SE	*P* value
Coffee intake	rs4410790	7:17284577	*AHR*	C/T	0.632	0.0391	0.0017	1.20 × 10^−120^
Coffee intake	rs2472297	15:75027880	*CYP1A1-CYP1A2*	T/C	0.263	0.0465	0.0018	1.10 × 10^−142^
Plasma caffeine	rs4410790	7:17284577	*AHR*	C/T	0.631	−0.0631	0.0087	4.03 × 10^−13^
Plasma caffeine	rs2472297	15:75027880	*CYP1A1-CYP1A2*	T/C	0.236	−0.0668	0.0104	1.50 × 10^−10^
eGFRcre	rs4410790	7:17284577	*AHR*	C/T	0.630	0.0023	0.0004	1.92 × 10^−10^
eGFRcre	rs2472297	15:75027880	*CYP1A1-CYP1A2*	T/C	0.260	0.0039	0.0004	8.21 × 10^−20^

AF, allele frequency; Chr, chromosome; eGFRcre, serum creatinine-based estimated glomerular filtration rate; SE, standard error.

a Position is based on CRGh37.

b A1 indicates effect allele, while A2 for another allele.

## Discussion

Based on GWAS results from East Asian and European ancestries, we estimated causal relationships of coffee consumption and caffeine levels with kidney function (eGFRcre) by applying different MR methods. We found that higher coffee intake was causally associated with higher eGFRcre levels in both populations. Another finding was that genetically determined higher caffeine levels were associated with lower eGFRcre levels among both genetic ancestries.

Scientific evidence for the coffee-kidney association is still sparse and the results are controversial. The recent US longitudinal cohort study with 14,209 participants found a lower risk of CKD among those who drank coffee (even less than 1 cup per day) compared to those who never consumed coffee.^38^ On the other hand, a previous meta-analysis reported no significant association between coffee intake and CKD.^13^ This study includes 4 population-based observational studies from Italy, Korea, and Japan. For clinical studies, a meta-analysis of 12 studies involving 505,841 patients confirmed that coffee has dose-response associations with lower risks of kidney outcomes (incident CKD, end-stage kidney disease, and albuminuria).^39^ The biggest concern for observational studies regarding this relationship could be reverse causation. It is plausible that coffee consumption can change depending on an individual’s kidney function. Given this major limitation in observational studies, MR would be an optimal approach to estimate this relationship without this type of bias.

To date, 2 previous MR studies have addressed this causal relationship. Kennedy *et al.*^17^ initially examined causal relationships between coffee consumption and 3 kidney outcomes (eGFR, CKD, and albuminuria) using GWAS meta-analysis across different ancestries. This study selected 22 variants for IVs and suggested beneficial effects of coffee consumption on eGFRcre (*β* = 0.022; 95% CI = 0.013 to 0.032) and CKD (odds ratio = 0.84; 95% CI = 0.72 to 0.98). Another MR study was reported by Mazidi *et al.*^16^ using only European GWAS summary statistics. They selected 5 genetic variants as IVs for MR analyses and reported no causal relationship of coffee intake with eGFRcre (*β* = −0.0005; 95% CI = −0.0103 to 0.0093) and CKD (odds ratio = 0.98; 95% CI = 0.86 to 1.12). Followed by these previous studies, our study performed MR analyses using the largest GWAS results from an East Asian population and suggested a protective role of coffee intake on kidney function. This is one of the strengths of this study and increases its scientific validity for this causality. As an additional technical development, our analysis in East Asian populations is more convincing because there is no substantial heterogeneity. Kennedy *et al.*^17^ reported a protective effect of coffee intake on kidney health in MR analyses; however, their analysis in European populations could introduce a bias by large heterogeneity of the selected variants. Furthermore, our MR analysis can avoid weak instruments for coffee consumption, whereas previous studies raised this problem as one limitation. In our analysis, by applying rigorous criteria for selecting IVs (*P* < 5.0 × 10^−8^; *F*-statistic > 10; linkage disequilibrium: *r*^*2*^ < 0.01), the results might not be biased by weak or correlated instruments.

Caffeine (1,3,7 trimethylxanthine) is one of the well-known chemical substances contained in coffee. Caffeine is associated with various health outcomes, including cardiovascular disease and diabetes.^29^^,^^40^ For genetic liability of blood caffeine levels, Cornelis *et al.*^41^ performed a GWAS for several caffeine metabolites among 9876 European individuals. They identified several variants within 4 possible genomic regions associated with caffeine metabolites. Of which, alleles at rs4410790 (*AHR*; C) and rs2472297 (*CYP1A2;* T) were associated with lower plasma caffeine levels and higher paraxanthine-to-caffeine ratio. This finding suggest that effect alleles at these 2 variants may play a role in faster caffeine metabolism compared with the alternative allele for each variant. Interestingly, many previous GWAS studies on coffee or caffeine intake have already shown that the effect alleles of rs4410790 and rs2472297 are associated with higher coffee intake. This phenomenon can be explained because people with faster caffeine metabolism can drink more coffee than those who can metabolize caffeine slowly. Based on this evidence, inconsistent directions of estimates from our MR analyses between coffee-kidney and caffeine-kidney associations are just derived from genetic variants.

There are several limitations to be mentioned. Although our MR analyses showed a protective effect of coffee on kidney function and an inverse association of caffeine levels with kidney function, interventional investigations should be applied to confirm this causal relationship. We found a substantial heterogeneity even after excluding genetic IVs resulting in pleiotropy. Heterogeneity could be due to additional pleiotropy, but other sources should be considered such as example canalization, gene-environment interactions, or population stratification.^15^ However, it is difficult to distinguish which resources cause tests for heterogeneity to be significant. Therefore, the results are possibly biased and should be carefully interpreted. In addition, our MR analysis addressed a linear association between coffee consumption and eGFRcre. Meanwhile, it is more worthful to investigate nonlinear association using both observational design and MR approach. We incorporated caffeine levels to understand metabolic backgrounds behind the coffee-kidney relationship. However, other noncaffeine metabolites and possible biological mediators, particularly obesity, hypertension, and diabetes, should be included in MR framework.

In conclusion, higher coffee consumption was associated with higher eGFR levels in East Asian and European ancestries, but caffeine levels in blood showed a negative causal association with eGFRcre. This result heavily relies on the genetic variants used for MR analyses, and the association with noncaffeine metabolites needs to be further investigated. As with this study, MR studies on coffee intake and caffeine levels will require careful interpretation.

## Disclosure

All the authors declared no competing interests.

## Supplementary Material

Supplementary File (PDF)

**Figure S1.** Scatter plot for the SNPs associated with coffee intake against SNPs associated with eGFRcre among East Asian ancestry using 11 original SNPs. Vertical and horizontal grey-colored solid lines around points show 95% CI.

**Figure S2.** Leave-one-out MR estimates of genetically-estimate coffee intake on eGFRcre among East Asian ancestry using 11 original SNPs.

**Figure S3.** Forest plot for effect size of genetically-estimate coffee intake on eGFRcre among East Asian ancestry using 11 original SNPs.

**Figure S4.** Leave-one-out MR estimates of genetically-estimate coffee intake on eGFRcre among East Asian ancestry using 7 rigorous SNPs.

**Figure S5.** Forest plot for effect size of genetically-estimate coffee intake on eGFRcre among East Asian ancestry using 7 rigorous SNPs.

**Figure S6.** Scatter plot for the SNPs associated with coffee intake against SNPs associated with eGFRcre among European ancestry using 28 original SNPs. Vertical and horizontal grey-colored solid lines around points show 95% CI.

**Figure S7.** Leave-one-out MR estimates of genetically-estimate coffee intake on eGFRcre among European ancestry using 28 original SNPs.

**Figure S8.** Forest plot for effect size of genetically-estimate coffee intake on eGFRcre among European ancestry using 28 original SNPs.

**Figure S9.** Leave-one-out MR estimates of genetically-estimate coffee intake on eGFRcre among European ancestry using 22 rigorous SNPs.

**Figure S10.** Forest plot for effect size of genetically-estimate coffee intake on eGFRcre among European ancestry using 22 rigorous SNPs.

**Table S1.** List of summary dataset for MR analysis in East Asian and European ancestries.

**Table S2.** MR-estimated effect sizes of coffee-kidney function associations in East Asian and European ancestries using original genetic variants.
