# Supplementary material for: Coffee Intake, Plasma Caffeine Levels, and Kidney Function: Two-Sample Mendelian Randomization Among East Asian and European Ancestries
Source: Kidney Int Rep. 2024 Jan 19;9(4):1083–92. doi: 10.1016/j.ekir.2024.01.024 (PMC11101828; doi:10.1016/j.ekir.2024.01.024)
Supplement: Supplementary File (PDF) [file mmc1.pdf]

Coffee intake, plasma caffeine levels, and kidney function: two-sample Mendelian randomization among East Asian and European ancestries

Ryosuke Fujii[1,2,3*], Masahiro Nakatochi[4], Fabiola Del Greco M.[1]

Contents:

Supplementary Tables S1-S2 -- **P.2-3**

Supplementary Figures S1-S10 -- **P.4-13**

Supplementary Table S1. List of summary dataset for MR analysis in East Asian and European ancestries

Ancestry	Association	Traits	N	Unit	Study	Overlap	Information
East Asian	SNP-Exposure1	Coffee intake frequency	152,634	A cup of coffee intake per day	BBJ	None	https://pheweb.jp/pheno/Coffee
	SNP-Exposure2	Plasma caffeine levels	8,940	1-SD increase in unit of metabolites	ToMMo	None	https://jmorp.megabank.tohoku.ac.jp/gwas-analyses/TGA000005-abbd47ef
	SNP-Outcome1	eGFRcre	47,070	1-SD increase in eGFRcre	ToMMo	None	https://jmorp.megabank.tohoku.ac.jp/gwas-analyses/TGA000007-a1873f7c
	SNP-Outcome2	eGFRcre	154,633	1-SD increase in eGFRcre	BBJ	None	https://pheweb.jp/pheno/eGFR
European	SNP-Exposure1	Coffee intake frequency	428,860	A cup of coffee intake per day	UKBB	None	https://gwas.mrcieu.ac.uk/datasets/ukb-b-5237/
	SNP-Exposure2	Plasma caffeine levels	7,719	1-SD increase in unit of metabolites	KORA TwinsUK		https://www.ebi.ac.uk/gwas/studies/GCST90243411
	SNP-Outcome	eGFRcre	567,460	1 increase in ln(eGFRcre)	CKDGen	None	https://ckdgen.imbi.uni-freiburg.de/files/Wuttke2019/20171017_MW_eGFR_overall_EA_nstud42.dbgap.txt.gz
		eGFRcys	24,063	1 increase in ln(eGFRcys)	CKDGen	None	https://ckdgen.imbi.uni-freiburg.de/files/Gorski2017/CKDGen_1000Genomes_DiscoveryMeta_eGFRcys_overall.csv.gz

Supplementary Table S2. MR-estimated effect sizes of coffee-kidney function associations in East Asian and European ancestries using original genetic variants.

	East Asian (11 SNPs)		European (28 SNPs)	
	Beta (95% CI)[1]	*P*-value	Beta (95% CI)[2]	*P*-value
Inverse-variance weighted	-0.105 (-0.242, 0.033)	0.13	0.027 (–0.009, 0.063)	0.14
Weighted median	0.101 (0.039, 0.163)	0.002	0.060 (0.043, 0.077)	7.02×10^{-12}
Weighted mode	0.112 (0.051, 0.174)	0.005	0.064 (0.051, 0.077)	2.59×10^{-10}
MR-Egger	-0.192 (-0.431, 0.047)	0.15	0.091 (0.026, 0.156)	0.01
Heterogeneity (IVW)	Q=193.58	3.51×10^{-36}	Q=435.96	2.26×10^{-75}

[1]One-standard deviation change in eGFR per coffee intake per week

[2]One-unit change in In(eGFR) per a cup of coffee per day

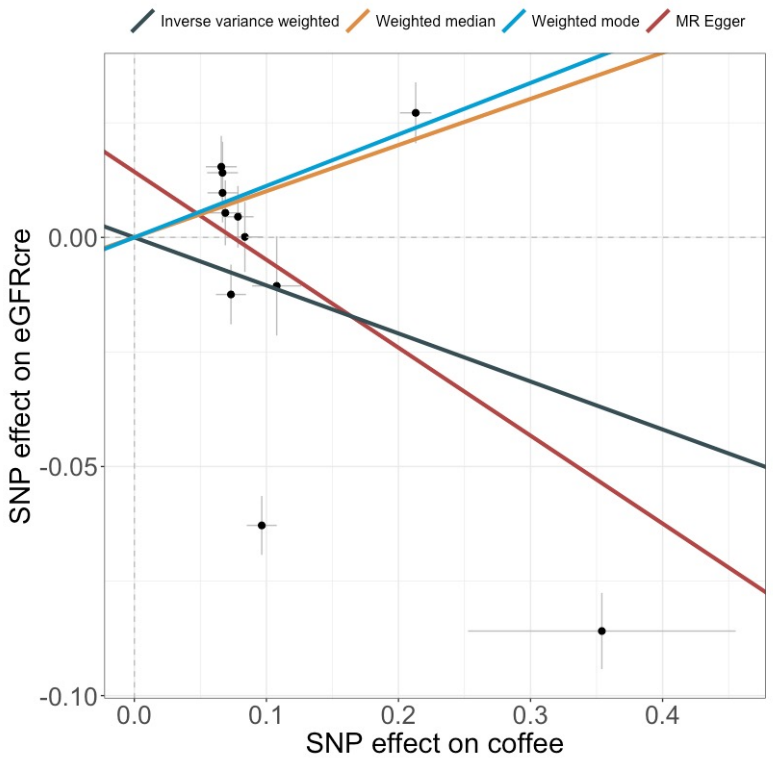

Figure S1. Scatter plot for the SNPs associated with coffee intake against SNPs associated with eGFRcre among East Asian ancestry using 11 original SNPs. Vertical and horizontal grey-colored solid lines around points show 95% CI.

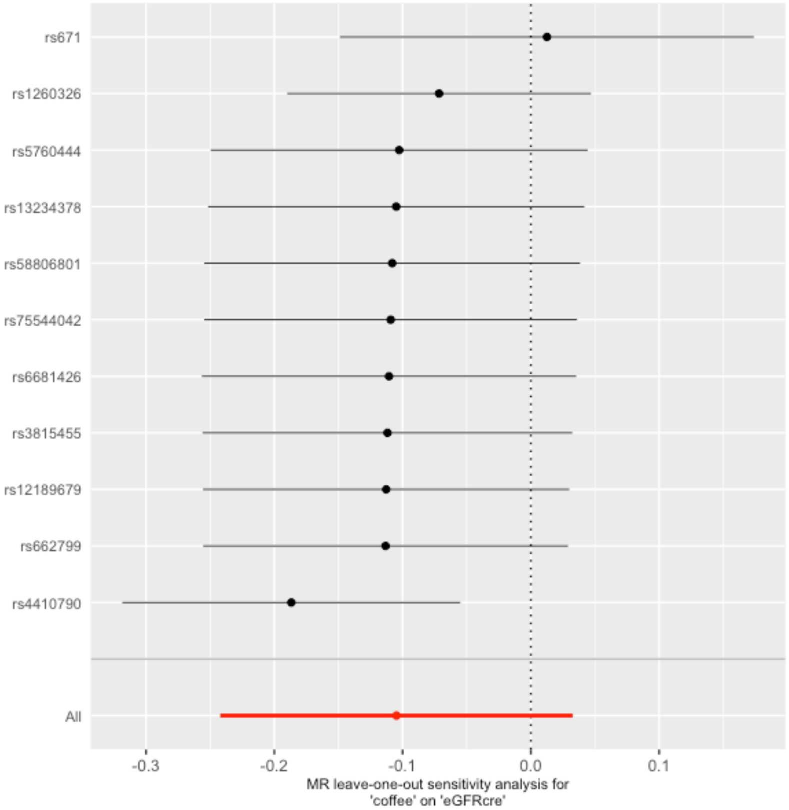

Figure S2. Leave-one-out MR estimates of genetically-estimate coffee intake on eGFRcre among East Asian ancestry using 11 original SNPs

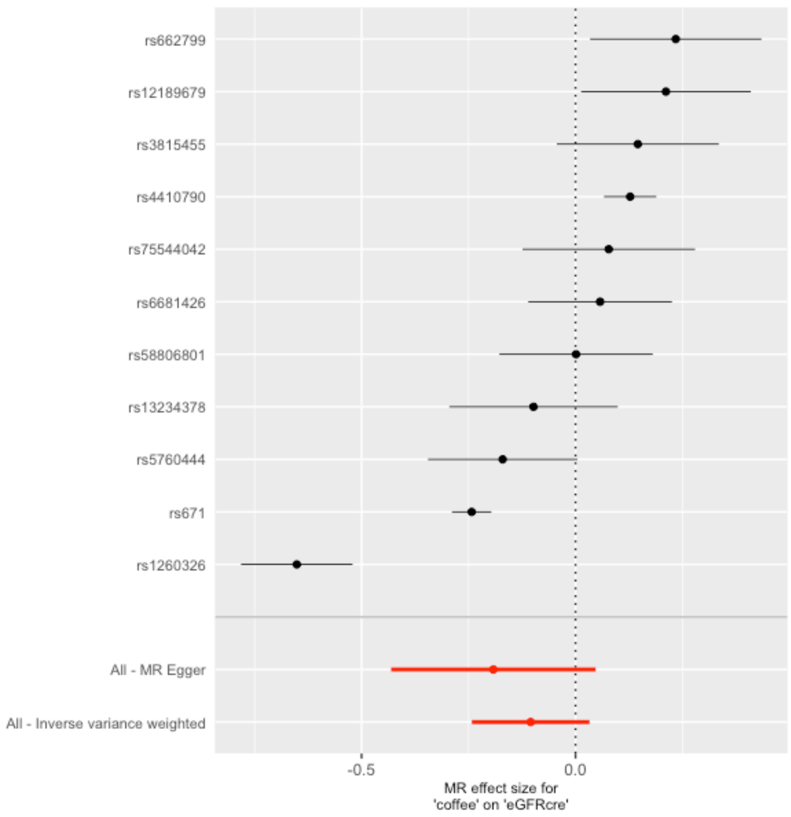

Figure S3. **Forest plot for effect size of genetically-estimate coffee intake on eGFRcre among East Asian ancestry using 11 original SNPs**

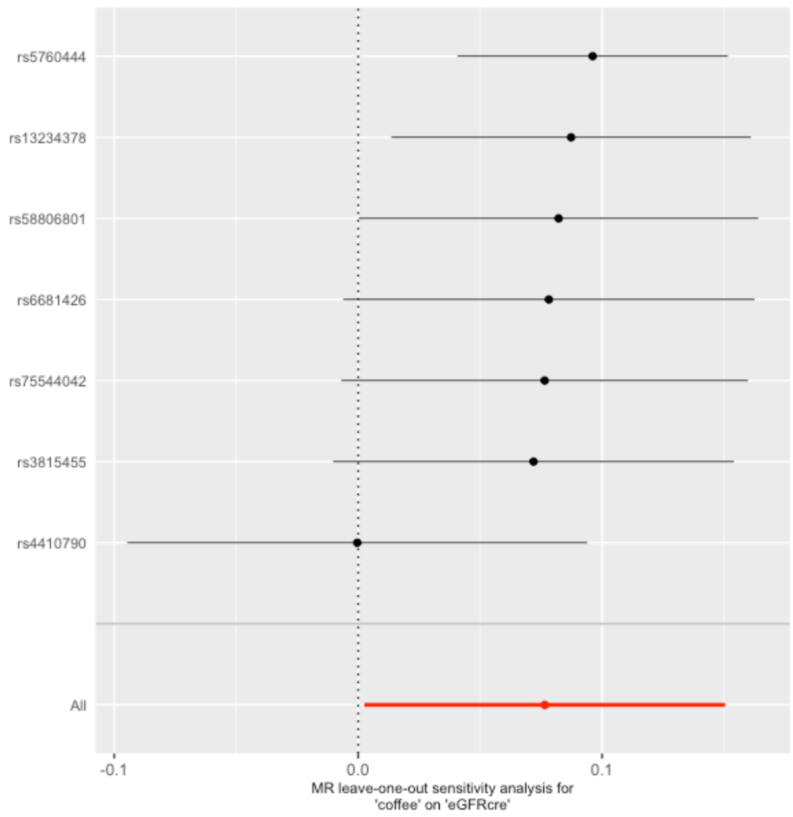

Figure S4. **Leave-one-out MR estimates of genetically-estimate coffee intake on eGFRcre among East Asian ancestry using 7 rigorous SNPs**

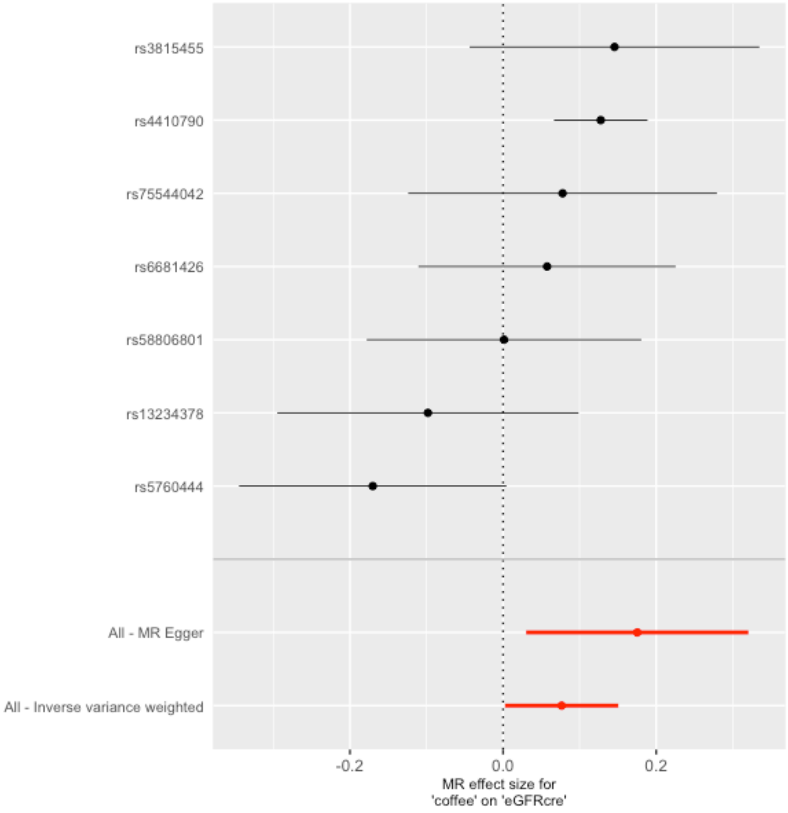

Figure S5. **Forest plot for effect size of genetically-estimate coffee intake on eGFRcre among East Asian ancestry using 7 rigorous SNPs**

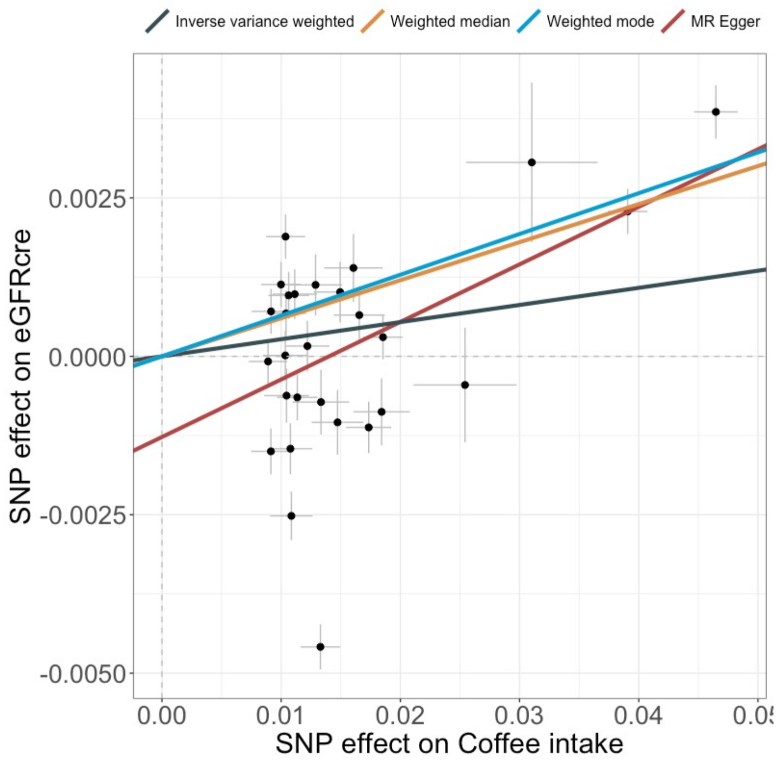

Figure S6. Scatter plot for the SNPs associated with coffee intake against SNPs associated with eGFRcre among European ancestry using 28 original SNPs. Vertical and horizontal grey-colored solid lines around points show 95% CI.

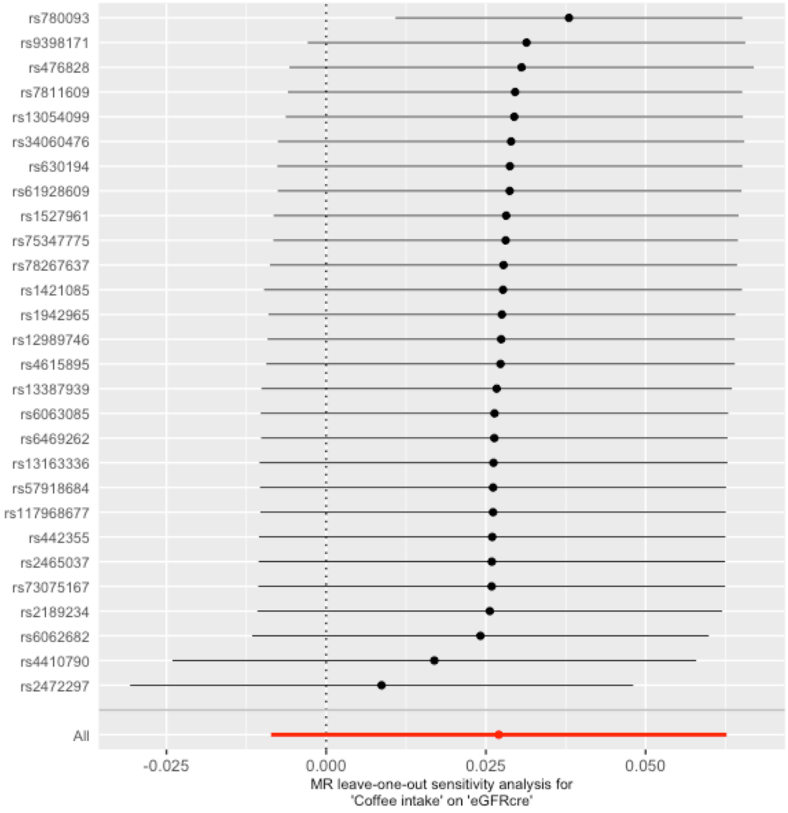

Figure S7. Leave-one-out MR estimates of genetically-estimate coffee intake on eGFRcre among European ancestry using 28 original SNPs

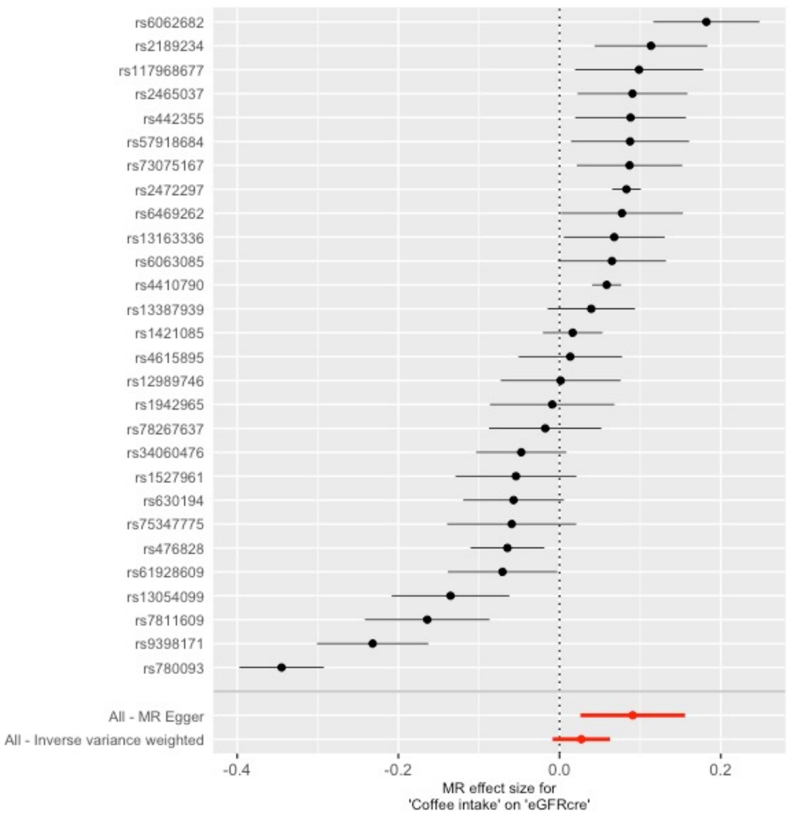

Figure S8. **Forest plot for effect size of genetically-estimate coffee intake on eGFRcre among European ancestry using 28 original SNPs**

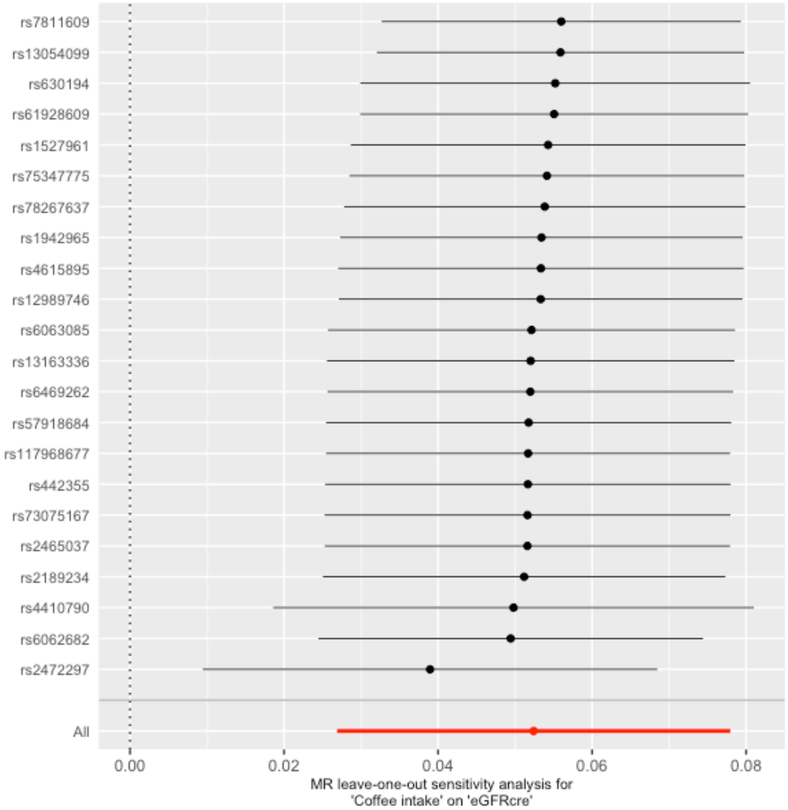

Figure S9. **Leave-one-out MR estimates of genetically-estimate coffee intake on eGFRcre among European ancestry using 22 rigorous SNPs**

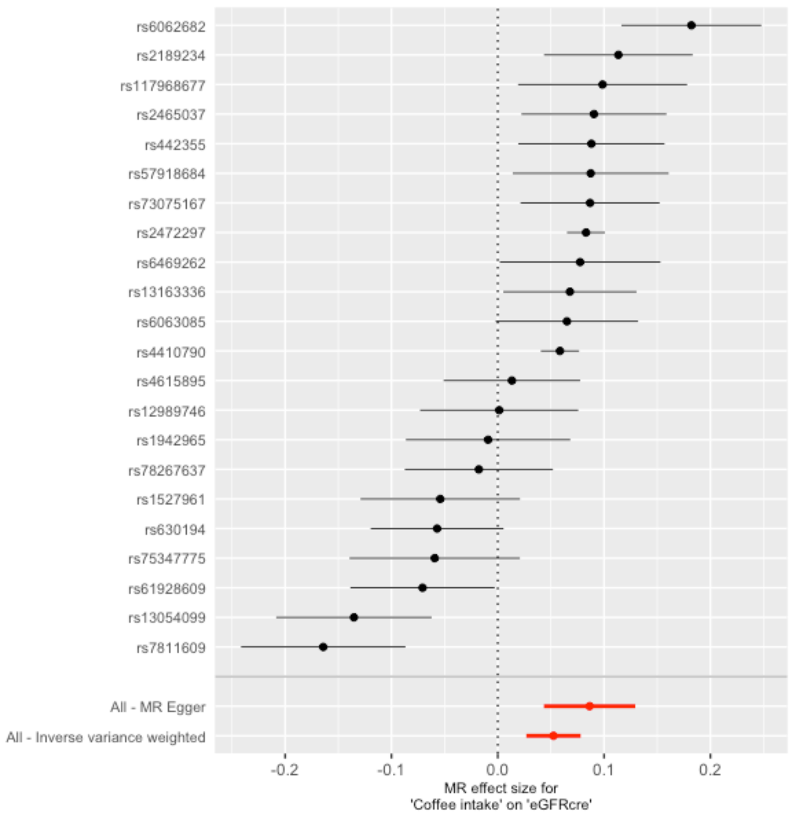

Figure S10. **Forest plot for effect size of genetically-estimate coffee intake on eGFRcre among European ancestry using 22 rigorous SNPs**
